# Are We Faced with Two Human Species?

**DOI:** 10.1100/tsw.2004.192

**Published:** 2004-11-02

**Authors:** Mladen Davidovic

**Affiliations:** Geriatric Clinic KBC Zvezdara, 11050 Beograd, Rifata Burdzevica 31, Serbia

**Keywords:** aging, interindividual variability, genetic stability, elderly, population groups

## Abstract

Problems could be found in the fact that we very often look for one deciding, definitive reason for the process of aging. It is a sort of search for a big discovery, like a fountain of youth or such. More and more authors are trying to explain the unknowns in the understanding of these observations about aging by adding the statement that there are two subgroups in the general population. This acknowledgment of two subpopulations explains why there are numerous cases that cannot be explained, defined, or fitted in basic observations about caloric restrictions and the delay of reproduction. The identification of those two groups would allow us to find more realistic results in studies and therefore a more efficient therapy of certain diseases. This hypothesis does not contradict theories of aging that we have accepted (at least not the majority of accepted theories), and this hypothesis also does not contradict the fact that there is a large interindividual variability. This hypothesis doubts, and claims there are exceptions to, the starting assumption of geriatrics and gerontology that: “parallel to the aging process the functions of all organs and organ systems lessen. ”

## INTRODUCTION

Nature seems to be cyclical in its essence; it appears to act as a sum of births, existences, and deaths of its parts. Human civilization seems to follow that same cycle of existence. However, just like the individuals in all other species, every one of us is stained by our instinct for survival. Further, in human beings, maybe due to our mental capacities, this survival instinct has translated into efforts to achieve longevity. The quest for longevity has been an integral part that has permeated the way we think and act. Magic, medicine, and religion have all played a role in this quest.

The way medicine has approached this issue has been to deal with symptoms. Cancers, strokes, and heart attacks are still the number one cause of death or low quality of life. We need to change the way we look at longevity and old age. As long as humans have been around, we attempted to find a "cure" for old age, find some kind of fountain of youth, and although we have improved, our most meaningful accomplishments did not come from treating symptoms, but from preventing illnesses.

The introduction of vaccination has prolonged human life by 28 years on average. So would it be possible to find a "vaccine" for the prevention of old age? Problems could be found in the fact that we very often look for one deciding, definitive reason for the process of aging. But as always, in life or medicine, the causes of aging are made up of many factors and can only be differentiated by their priorities. On the other hand, to try to simplify old age by saying that it is the same as middle age, except for the effect of survival of the fittest, would be oversimplifying the issues.

## DISSCUSION

If we tried to sum up everything we know today about aging into two sentences, as much as such simplifications can be dangerous, we would probably come up with the following two sentences or basic observations:

1. A calorie-restricted diet does prolong life
2. Delay of the reproductive processes also prolongs life[1,2].

More and more authors are trying to explain the unknowns in the understanding of these observations about aging by adding the statement that there are two subgroups in the general population. Such a statement does not contradict the basic observations listed here. This acknowledgment of two subpopulations explains why there are numerous cases that cannot be explained, defined, or fitted in these basic observations about caloric restrictions and the delay of reproduction.

The rate of aging is influenced by many different genes. Recent studies[3,4,5], together with previous studies[6,7], provide the "glue " between the two underlined basic observations. First, the mutation of genes that prolong life for *C. elegans* conditions certain changes in the metabolism, for example, leading to eating disorders. In addition, the mutation influences the changes in the levels of regulatory proteins, also playing a role in the metabolism (daf-2/insulin-like growth factor-IGF1). DAF-16 regulates the response for oxidative stress.

Second, mutations in certain genes prolong life. It seems then that those genes in "normal" conditions actually play a role in the shortening of a lifespan for an individual. The key is their function. They allow the reproductive advantages for the species or for the individual. The mutation of these genes prolongs life, but lessens reproductive capability.

The two known options for the prolongation of life, listed here as basic observations about aging, are applicable to the majority of the general population. The rest of the population, as much as it might seem awkward, can be considered as privileged. The primary group, i.e., the majority of the population, are those that have evolved successfully; in other words, there would be no evolution if it was not for this group. The privileged group, "the selfish ones", are those that are genetically more stable. If the privileged group was the majority, the human race would not have gone down the same evolutionary road (Fig. 1).

More often, we hear authors of studies speak of two subpopulation groups. One group that ages by all the laws of aging that we have encountered and excepted thus far and the other group that seems to postpone aging[8] due to "programmed death" or more specifically due to low mastery/low emotional support[9] or because of additional reasons, but the existence of two groups seems evident[10].

The identification of those two groups would allow us to find more realistic results in studies and therefore a more efficient therapy of certain diseases. This hypothesis does not contradict theories of aging that we have accepted (at least not the majority of accepted theories), and this hypothesis also does not contradict the fact that there is a large interindividual variability. This hypothesis doubts, and claims there are exceptions to, the starting assumption of geriatrics and gerontology that: "parallel to the aging process the functions of all organs and organ systems lessen." This hypothesis also puts doubt to the belief that elderly of 85 or above are necessarily dependent on help and care of others. Some are, some are not. The fact that the first group is probably larger does not and should not affect the claim about the existence of two groups. The reason for this is not exclusively large heterogeneity, but also the existence of two subpopulation groups. If the reason for all the differences was in the interindividual variability, then those differences would exist in all the other groups of elderly, but they exist in the group of the most elderly (85 and above) as compared to all other groups of elderly. Therefore, the linear decline is found in groups from 65–69, 70–75, 76–80, but it is not necessarily found in individuals that are even older, since these individuals are more easily compared to and closest to the ages of 55–65.

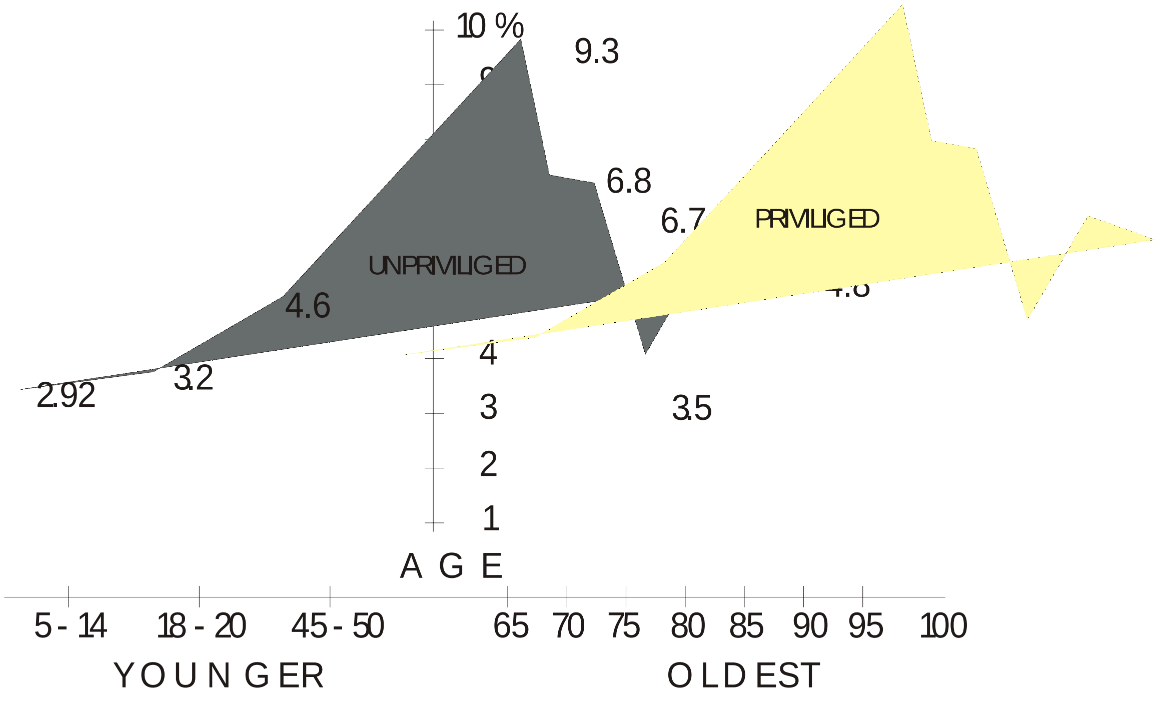

**FIGURE 1**. The rise of the chromosome aberrations in the “privileged” group is hypothetically delayed[8].

The fact is that in research studies, the group of elderly on average is not older than 60 years. The group of the oldest is usually from one cohort[11,12,13,14]. This ends up meaning that the whole group is made up of those dependant on help and care of others (homes for the aged) or from those “top-fit” elderly that are willing and have the energy to participate in investigative research studies. It should not be validated that all the differences in the group end up being only interindividual, which is what happens in most cases. Based on studies so far, this happens in the 65–80 group. The most elderly group does differ in many ways in studies done so far, and this difference is more pronounced than the differences between other groups of elderly!

A large part of the research dedicated to the field of gerontology has indicated that the people who live long are indeed a privileged group. The differences in the two subpopulation groups of elderly often end up being about “choices”, which leads to primary differences.

It is very possible that, as often happens in medicine and biology, the difference is the result of more than one cause, both genetic and environmental[15,16,17,18,19,20,21,22,23,24]. If there is such a split in the elderly population, a split into subpopulations, then the results of that fact are not only theoretical. This can be the reason for differences in results of studies whose concepts are parallel. This understanding can lead to changes of programs and institutions that seem to have been based on misconceptions and therefore are not successful, or are not successful enough in treating the elderly.

We should consider the hypothesis that there are people with more-stable genomes and people with less-stable genomes. Identification of those two groups of individuals would allow us to “vaccinate” and through that, act on the instable genomes and genes. By that we would be able to prevent the development of cancer, for example. Working under the assumption that cancer will, after enough chances for mutation, be sure to develop, then the two groups of individuals we are discussing are those with less-stable genomes (whose genes react faster to mutation) and those with more-stable genomes (whose genes react more slowly to mutation). If we were able to prevent or at least prolong the onset of malignant developments or cardiovascular diseases, we would be able to prolong human life in a significant manner.

Thus, medical history has taught us that if we truly want to be effective, we must not only treat symptoms, although at times that is the best we can do, we must also introduce preventive measures. Old age should be seen as something that can be prevented or postponed significantly as well.

The fact is that the knowledge we have now about aging tells us that activity and healthy food are all crucial for a healthy life. However, how many people actually follow this prescription? The effect of these preventive measures is minimal today, however, the effect of a “vaccine” would be broader and would cut across population lines. The whole human population could benefit from such an approach.

This attempt to deal with aging should not imply that old age cannot be beautiful and meaningful, but that prolonging life can allow human beings to accomplish more and achieve that dream of longevity. Critics might question to what extent it is up to us to prolong life, to what extent it is up to us to change the course of nature… and that is a reasonable concern. However, the concept of a “longevity vaccine” should be seen as a tool to improve quality of life, not to alter it. Further, as of now, we still do not have this tool, so although it is reasonable to think about its ethical implications, we must also think about the ethical implications of not developing something that could improve the life of so many, without discrimination.

Further criticism might be directed at the far-reaching effects behind such an endeavor in terms of population growth and size. It is obvious that there are problems with overpopulation of the planet, but in no way can we morally allow ourselves to attempt to deal with overpopulation by not offering the best medical services we can. Further, we should ask ourselves how would this world have looked like if __________ had lived longer.

## CONCLUSION

It seems obvious that the phenomenon of aging as related to the survival of the species is only important to humans, who are always fighting for extensions; in this case, extension of life.

Further, it seems that caloric restrictions are the only somewhat documented way of prolonging human life, as documented in experiments done on animals.

It is a matter of fact that everyone points to a genetic basis of longevity as crucial, yet the number of suggested explanations and definitions for the role of genetics in longevity is unacceptably large. It might be time to accept that which we already know and that is that genetic stability is the basis of longevity and further that there exist two population groups regarding this differential. Most of the suggested definitions and explanation of the genetic basis behind longevity are focused on the mechanism of this correlation and they are mostly all relevant to the final explanation of this puzzle. It is only natural that all the authors claim that the most important part of the puzzle is the very subject they examine.

It is undisputable that relatives of the long-lived live longer as well. The question that arises is whether these relatives are also genetically more stable compared to the rest of the population. The implied premise here is that the long-lived are, in fact, genetically more stable then the rest of the population[ 8].

What we do know is that the healthy elderly exhibit the same replicative capacity of cells as younger adults[25].

Genetic instability is an absolute advantage for a species in an evolutionary sense. The species that are more likely to adapt obviously are more likely to survive in a changing environment. On the other hand, a smaller amount of organisms with stable genetic material are more likely to have a long life. In any case, their cells are also affected by negative environmental conditions causing oxidative damage, but their genetic material is more resistant, i.e., less susceptible so there is a smaller percentage of illnesses. These organisms are not immortal (who truly wants that after all?), but they are less susceptible to illnesses.

It seems that the right thing to do now would be to try to examine to what extent the cells of the relatives of the long-lived are stable, both absolutely and relatively, in relation to the rest of the population using standard methodology[26,27].
